# Identification of Activation Isotopes in a CS-30 Cyclotron Vault

**DOI:** 10.3390/s22072581

**Published:** 2022-03-28

**Authors:** Alhussain A. Abuhoza, Hamoud A. Kassim, Ahmed A. Alghamdi, Faisal M. Alrumayan, Mehenna Arib, Ibrahim J. Aljammaz, Meshari ALQahtani

**Affiliations:** 1 Nuclear Science Research Institute, King Abdulaziz City for Science and Technology (KACST), Riyadh 11442, Saudi Arabia; aabuhoza@kacst.edu.sa (A.A.A.); ahmedg@kacst.edu.sa (A.A.A.); 2 Physics and Astronomy Department, College of Science, King Saud University (KSU), P.O. Box 145111, Riyadh 11362, Saudi Arabia; hkassim@ksu.edu.sa; 3 King Faisal Specialist Hospital and Research Centre, P.O. Box 3354, Riyadh 11211, Saudi Arabia; rumayan@kfshrc.edu.sa (F.M.A.); marib@kfshrc.edu.sa (M.A.); jammaz@kfshrc.edu.sa (I.J.A.)

**Keywords:** gamma spectrometry, high-purity germanium (HPGe), Monte Carlo simulation, radionuclides, dose rate

## Abstract

A CS-30 cyclotron has been in operation at King Faisal Specialist Hospital and Research Center (KFSHRC) since 1982. The CS-30 cyclotron has been used to produce medical radioisotopes for positron emission tomography (PET) and single-photon emission computed tomography (SPECT). Some of the nuclear reactions of radionuclide production are associated with the intense release of a wide range of fast neutrons. In this work, we investigated the radionuclides produced from neutron interactions with the cyclotron facility walls. Activation isotopes were determined by performing gamma ray spectrometry utilizing a high-purity germanium (HPGe) detector. The major radionuclides found were ^152^Eu, ^154^Eu, ^134^Cs, ^65^Zn and ^60^Co. Activation isotope accumulation had increased the dose rate inside the facility. The surface dose rates were measured at all of the surrounding walls. The maximum surface dose rate was found to be 1.2 µSv/h, which is much lower than the permissible occupational exposure of 15 µSv/h based daily 5 work hours.

## 1. Introduction

Particle accelerators, such as cyclotrons for medical isotope production, are usually surrounded by thick concrete structures [1,2]. Most medical cyclotrons have been used to perform (p, n), (p, α) and (d, 2n) reactions to produce radionuclides [3]. The generated alpha particles had low penetrating power, while the produced neutrons penetrated the air and struck the walls of the cyclotron room. Then, neutrons induced radioactive nuclides in the inner concrete wall through several fast and thermal neutron interactions [4,5,6,7,8]. The accumulation of radionuclides in the cyclotron facility walls raised the occupational exposure of workers. In addition, concrete wall activation might lead to an issue of radioactive waste management in the case of facility decommissioning [9,10,11].

Several experimental and modeling studies have investigated fast- and thermal-neutron-induced isotopes using gamma spectroscopy detection methods. Martínez-Serrano et al. [12] predicted the activity levels of several neutron-induced radionuclides in the wall of the cyclotron facility at the University of Malaga using MCNPX code [13]. The calculation of radioactivity as a function of wall depth revealed the presence of ^152^Eu, ^154^Eu, ^60^Co, ^134^Cs, ^46^Sc, ^54^Mn and ^65^Zn. Furthermore, Kambali et al. [14] utilized gamma spectroscopy using a NaI(Tl) detector in addition to TALYS code to determine the activation radionuclides of the CS-30 cyclotron of the BATAN center. The study stated that the major isotopes observed in the measured gamma spectrum and in accordance with the simulation were ^60^Co and ^134^Cs. Yamaguchi and coworkers [15] detected several neutron-induced radionuclides, namely ^46^Sc, ^60^Co, ^65^Zn, ^134^Cs, ^152^Eu, ^22^Na and ^54^Mn, in the medical compact cyclotron room at Gunma University Hospital. In addition, the study showed that the neutron capture (n,γ) reaction was the predominant neutron reaction with wall elements, and that radionuclide activity decreased exponentially through the wall depth. Fujibuchi et al. [16] performed gamma spectrometry using a HPGe detector on several concrete samples taken from the cyclotron room. Only two distinct long-lived radionuclides, ^60^Co and ^152^Eu, were identified, since the measurements were taken two years after shutting down the cyclotron. 

S. Vichi et al. [17] implemented a Monte Carlo simulation code and validated a simulation by experimental work to evaluate the activation of bunkers at the S. Orsola-Malpighi Hospital in Italy and Bern University Hospital at Switzerland. The most active radionuclides in the concrete walls of the two bunkers were revealed to be ^46^Sc, ^60^Co, ^65^Zn, ^134^Cs, ^152^Eu, ^22^Na and ^54^Mn. Radionuclide activities were found to be within the range of 0.01 to 2 Bq/g. Most observed radionuclides had a good correlation between Monte Carlo results and experimental measurements, except for ^152^Eu, ^46^Sc and ^65^Zn.

In a recent article, Go Yoshida et al. [18] performed an accelerator decommissioning study by conducting a series of surveys in synchrotron radiation facilities in Japan to evaluate the activation of walls and accelerator beam components. Various dosimeters were used to measure ambient neutron flux at various locations of the facilities. It was found that thermal neutron fluxes ranged between 10^0^ and 10^2^ cm^−2^s^−1^, corresponding to activities of 10^−5^ and 10^−3^ Bq·g^−1^. In addition, the maximum measured dose rate was found to be 5 µSv/h.

In this study, the activation isotopes in the CS-30 cyclotron vault walls at KFSHRC were investigated and identified utilizing an HPGe detection system. The gamma spectrum was analyzed thoroughly by considering the interacting neutron energies and vault wall elements. Furthermore, a Geant4 simulation was conducted to obtain the generated neutron spectrum from the proton beam bombardment of the CS-30 internal and external targets. Through a combinatory analysis of simulations, chemical analysis of the wall concrete and gamma ray spectrometry measurements, 10 radionuclides were identified. Finally, surface dose rates were measured in four locations to identify the maximum dose rate in the facility.

## 2. Materials and Methods

### 2.1. The CS-30 Cyclotron Facility

**Figure 1:**
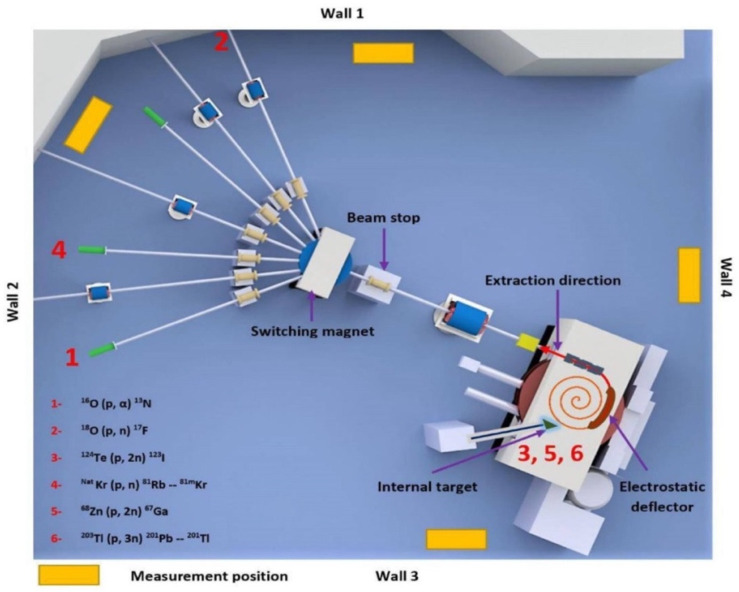
Layout of the CS-30 vault.

The CS-30 cyclotron at KFSHRC has been under full operation since 1982, producing PET and SPECT radioisotopes such as F-18 and Tl-201 [19]. The CS-30 is a positive ion machine capable of accelerating protons, deuterons, helium-3 and helium-4 of different energies. However, in the last three decades, a 26.5 MeV monoenergetic proton beam has primarily been used for the production of medical imaging isotopes. Currently, an IBA Cyclone 30 has been used as a major isotope production cyclotron at KFSHRC due to its high reliability, low exposure and dual-beam extraction capability [20]. However, the CS-30 is being kept as a backup cyclotron for producing medical isotopes in the case of a shortage because of its high running cost compared to the IBA Cyclone 30. The CS-30 cyclotron is housed in a 10 m long, 8 m wide and 6 m tall building with a wall thickness of 2.1 m. A 3D model of the CS-30 cyclotron and beam lines is shown in Figure 1.

The chemical composition of the concrete wall was analyzed using inductively coupled plasma mass spectrometry (ICP-MS) (model: ELAN 9000, PerkinElmer, Waltham, MA, USA). The ICP-MS sensitivity was measured by identifying the millions of counts per second (Mcps) per milligram per liter (mg/L), and it varied from one chemical element to another. For the PerkinElmer model 9000, the measurement sensitivity was greater than 30 Mcps/mg/L and 10 Mcps/mg/L for U and Mg, respectively. An ELAN 9000 model has a coefficient of variation (%RSD) of less than 3% RSD for a 10 µg/L multielement solution [21]. ELAN 9000 ICP-MS was utilized to analyze the chemical composition of vault wall 1. The results demonstrated that the wall was composed of elements commonly used in the construction of radiation shielding, such as Ca, Fe, Zn and Mg, although several other elements were also present in the solid mixtures, as listed in Table 1.

In the past four decades, CS-30 has been used to produce six PET and SPECT radionuclides (^13^N, ^18^F, ^123^I, ^81^Rb, ^67^Ga and ^201^Pb). The production of these radionuclides is associated with the release of neutrons due to proton interaction with targets and targets’ holding materials. Thus, the induced neutrons of the PET and SPECT production reactions require further investigation to identify the neutron spectra via Monte Carlo simulation code. All the produced radionuclides with the average beam time and beam current are listed in Table 2.

### 2.2. The Geant4 Model

**Figure 2:**
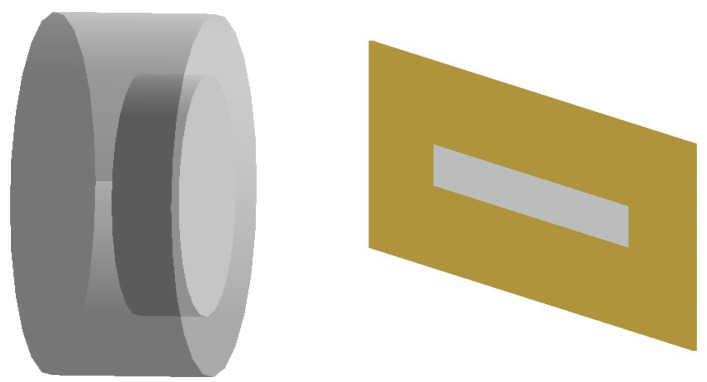
Geant4-simulated CS-30 targets: (**left**) external target and (**right**) internal target.

Geant4 is a powerful Monte-Carlo-based toolkit that simulates the passage of particles through matter [22]. Geant4 (version 10.05) code was built to determine the neutron spectra generated from the proton beam interaction with targets. Two types of CS-30 targets were simulated. The internal target consists of a 1 mm-thick copper plate. Furthermore, the internal targets (^124^Te, ^68^Zn and ^203^Tl) were electrodeposited on the copper plate (thickness = 0.25 µm). On the other hand, the external targets were simulated as aluminum capsules with a 3 cm radius and 2.4 cm thick. The inner target radius was simulated with a 2 cm radius and 1 cm thick and was placed 1 mm deep inside the capsule. The external targets were used for ^16^O, ^18^O and ^Nat^Kr isotopes. Figure 2 shows Geant4-simulated internal and external targets.

**Figure 3:**
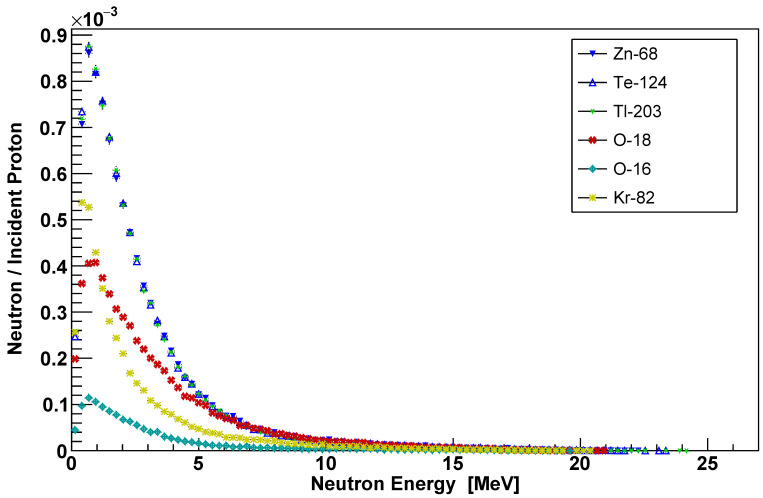
Neutron spectra produced from a simulated 26.5 MeV proton beam bombarding internal and external targets.

The proton beam was simulated as a monodirectional point source of 26.5 MeV with 10^6^ number of events. A binary cascade model was selected to simulate the passage of primary protons and secondaries through targets. In addition, a high-precision neutron package was included to transport fast neutrons down to thermal energies. The neutron spectra were recorded on the back-end of the targets. The simulated bombarding of targets with the proton beam resulted in wide neutron spectra, as shown in Figure 3.

The neutron yield had a significantly higher intensity for internal targets. Furthermore, all three internal targets almost have the same identical neutron spectrum, since all of the three targets have an extremely low thickness and hence the neutrons were released mainly from proton beam interactions with the holding copper plate. Nevertheless, the external targets showed varied neutron spectra. The ^16^O target has the lowest neutron intensity since released neutrons are not among the main nuclear reaction utilized to produce ^13^N. Furthermore, the neutron induced mainly from the proton beam interaction with the front thin layer of the aluminum capsule. Neutron spectra were generated through Geant4 simulation as guidance to perceive radionuclides out of neutron interactions in addition to the gamma spectrometry inside the CS-30 facility.

### 2.3. Gamma Spectroscopy System

The gamma-ray spectra from the vault wall were measured using a HPGe detector (model: GX4019, CANBERRA, Meriden, CT, USA) coupled with a Multi-Channel Analyzer (MCA) (model: 927, ORTEC, Oak Ridge, TN, USA). The system was calibrated using gamma button sources, namely ^60^Co and ^22^Na. The HPGe-obtained energy resolution (FWHM) was found to be 2.92 keV, or 0.25% at 1173 keV. The HPGe was placed in a perpendicular orientation to the wall at a height of 1.5 m off the floor, 0° relative to the proton beam direction and 0.25 m from the wall at four locations, as pinpointed in Figure 1. The gamma spectra were collected for 5 h for each position using 16K channel data memory. Furthermore, the surface dose rate of gamma radiations emitted from the walls of the cyclotron vault were measured using two different Geiger survey meters, calibrated at the secondary standard dosimetry laboratory of KFSHRC with a traceability to BIPM.

## 3. Results and Discussion

### 3.1. Activation Radionuclides

**Figure 4:**
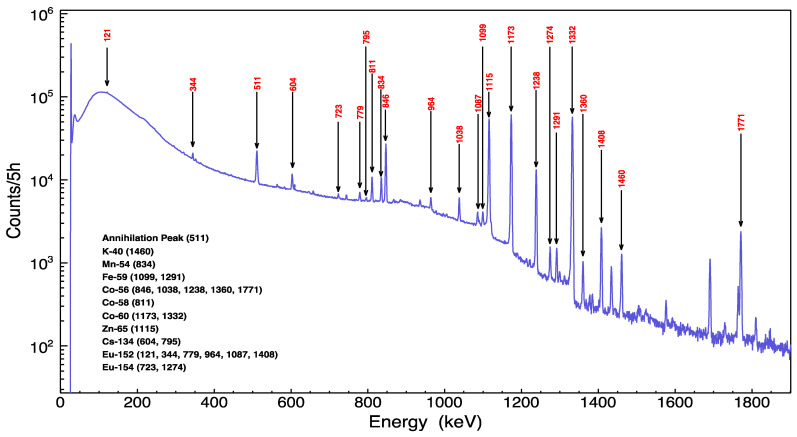
Gamma-ray spectrum of the CS-30 cyclotron vault wall 4.

Figure 4 illustrates the gamma spectra in the vicinity of location 4, which was pointed out in Figure 1. The most intense gamma peaks were analyzed thoroughly by considering the concentration of chemical elements in the wall and their natural abundance as well as potential neutron interactions based on cross-sectional data. For more sufficient analysis, any radionuclide with multiple gamma lines were investigated and advocated for by comparing the intensity of gamma lines with radionuclide abundant yields. Consequently, 10 radionuclides were determined as the byproducts of interactions of neutrons with wall elements. The radioisotopes were induced out of fast, thermal neutron and proton interactions.

^58^Co, ^54^Mn and ^40^K radionuclides were generated by fast neutron interactions. The ^58^Co isotope was produced by (n, 2n) and (n, p) reactions with cobalt and nickel elements, respectively, while ^54^Mn was generated through (n, p) interactions with iron. Furthermore, ^40^K is a natural radioactive isotope, but this work suggested that ^40^K was also created in the wall through neutron interactions with the ^40^Ca isotope. Calcium has the majority concentration in the wall, and most neutron interaction byproducts with ^40^Ca are stable isotopes. However, a ^40^Ca (n, p) ^40^K reaction produced a radioisotope and has a relatively high fast neutron cross-section as well.

On the other hand, the radioisotopes of ^152^Eu, ^154^Eu, ^134^Cs, ^65^Zn, ^60^Co and ^59^Fe were produced by thermal neutron capture reactions. ^152^Eu, ^154^Eu and ^134^Cs radioisotopes were observed clearly in the gamma spectra even with an insignificant amount of wall concentration due to their high neutron capture cross-sections. Nevertheless, ^65^Zn and ^60^Co have relatively high concentrations and neutron capture cross-sections. Furthermore, ^59^Fe was observed due to the high concentration of iron in the wall. However, ^59^Fe gamma lines had the lowest intensities, since the half-life of ^59^Fe is the lowest among the detected radioisotopes.

In contrast to all the recorded isotopes that were induced from direct neutron activation reactions, the ^56^Co radionuclide was produced from a (p, n) interaction with the ^56^Fe isotope. The interacted protons were induced from fast neutron (n, p) interactions inside the walls. Table 3 summarizes the nuclear reactions, half-lives, and the maximum neutron cross-sections with the respective neutron energies. Neutron cross-section values were obtained using the recent ENDF/B-VIII library [23]. Tabled cross-section values give an indication of radioisotopes’ production probability since the neutron spectra from the proton beam reaction with targets had a wide range of energies.

**Figure 5:**
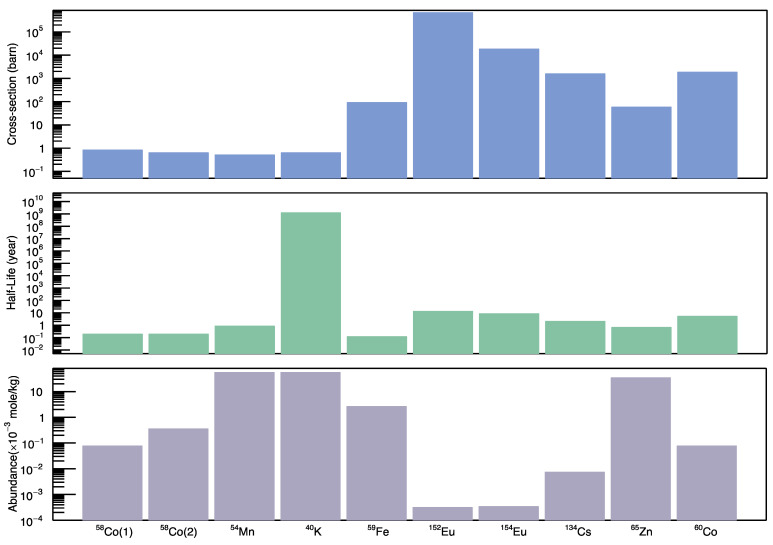
Maximum neutron cross-sections of nuclear reactions (**top**); half-lives of the isotopes (**middle**); and abundances of the radioactive isotopes in the wall (**bottom**).

To conclude, neutron cross-sections, half-lives and isotope abundances in the wall are the three main factors for radioisotopes’ presence. All three factors were plotted for all radionuclides except ^56^Co, as shown in Figure 5. Obviously, the produced radioisotopes mainly had either high neutron cross-sections, such as ^152^Eu and ^154^Eu, or relatively high abundance in the wall, as clearly seen for the other radioisotopes. However, the variation in the mentioned three factors in addition to the operation duration demonstrated the appearance and absence of isotopes in other CS-30 cyclotron facilities (e.g., Sc-46) [14]. Note that the existing CS-30 facility is in a cooling phase, and only used occasionally as a backup for SPECT isotope production.

### 3.2. Dose Rates Inside the CS-30 Vault

The measured dose rates of gamma radiation near the cyclotron vault walls were 0.96, 1.2, 1.0 and 0.6 µSv/h. The highest dose rate was recorded at wall 2, which is located near the target rooms and facing the beam line. In addition, wall 2 is the closest wall to the two of the external targets (^18^O and ^Nat^Kr). On the other hand, the lowest value was obtained at wall 4, which is located behind the beam line. There were slight differences between the measured dose rates due to the direction of the proton beam and wall to the target distance as well. The cyclotron surface dose rates were measured and had insignificant dose rate values.

Reducing the number of secondary neutrons would lead to a decrease in the dose rate inside the facility. Using a thick moderator (e.g., polyethylene) followed by a high thermal neutron cross-section material, such as boron, would thermalize and absorb generated neutrons, thus decreasing activation in the wall and reducing or maintain the dose rate inside the CS-30 facility. For future cyclotron facilities, Eu and Co elements must be refined from concrete compositions since both of them interacted with thermal neutrons and produced the most active and long-lived radionuclides, as shown previously in the literature [16], and their extremely high neutron cross-sections, as pointed out in Table 3.

## 4. Conclusions

The activation of the concrete wall of a CS-30 facility was investigated. CS-30 proton beam bombardment with a target generated a wide spectrum of neutron energies up to 26.2 MeV. The neutron flux was responsible for the wall activation over a long period of CS-30 operation. Fast neutrons interacted with walls, after which they slowed down and were absorbed through neutron capture reactions. The major radionuclides observed in the gamma spectrum and identified as a product of thermal neutron capture were ^152^Eu, ^154^Eu, ^134^Cs, ^65^Zn, ^60^Co and ^59^Fe. Furthermore, ^152^Eu and ^154^Eu have low abundances in the wall but both have high thermal neutron cross-sections, while ^134^Cs and ^60^Co have relatively high neutron cross-sections and abundances. ^65^Zn and ^59^Fe have high abundances and low neutron capture cross-sections. On the other hand, fast neutron interactions with the wall produced three radionuclides, namely ^58^Co, ^54^Mn and ^40^K. Furthermore, the fast neutron interactions produced recoil protons that interacted with ^56^Fe to originate ^56^Co.

The accumulation of radionuclides in the CS-30 facility walls lead to a slight increase in the dose rate. The dose rates from the four walls of the facility were measured using a Geiger survey meter. The maximum and minimum dose rates were 1.2 µSv/h and 0.6 µSv/h, respectively. The variation in the dose rates was due to the distance between the target location and proton beam direction. In order to minimize the wall activation, beam targets must be followed by thick neutron shielding with hydrogen content (e.g., polyethylene) and a high thermal neutron cross-section absorber (e.g., boron) to maintain or reduce the dose rate level of the facility. For low-activated concrete, the concrete composition must be purified from Eu and Co contents since both have extremely high neutron capture cross-sections and thus produce long-lived radionuclides with high activity.

## Figures and Tables

**Table 1:** Chemical composition of the CS-30 cyclotron’s concrete wall.

Element	Concentration	Element	Concentration (mg/kg)	Element	Concentration (mg/kg)
	(mg/kg)				
Li	12	As	17.5	Fe	53,139
B	1383	Rb	11.3	Co	4.6
Ne	437	Nb	1.6	Ni	31.1
Na	588	Mo	7.9	Cu	30.4
Mg	3788	Ag	2.2	Zn	4586
Al	2690	Cd	0.4	Ga	1.6
K	2326	Sn	26.4	Hf	0.3
Ca	152,501	Xe	3.9	Ta	0.4
V	18	Cs	1	W	1.8
Cr	48	Eu	0.1	Pb	102.8
Mn	675	Yb	0.12	U	0.9

**Table 2:** List of CS-30-produced PET and SPECT isotopes.

Nuclear Reaction	Average Beam Time per Year (h/Year)	Beam Current (µA)
^16^O (p, α) ^13^N	73	15
^18^O (p, n) ^18^F	1300	20
^124^Te (p, 2n) ^123^I	625	18
^Nat^Kr (p, n) ^81^Rb -- ^81m^Kr	390	30
^68^Zn (p, 2n) ^67^Ga	315	50
^203^Tl (p, 3n) ^201^Pb -- ^201^Tl	520	100

**Table 3:** The observed radionuclides and their nuclear reactions; cross-sections values were taken from the ENDF/VIII.0 neutron library.

Interaction Type	Activation Radionuclide	Nuclear Reaction	Half-Life (Years)	Maximum Neutron Cross-Section
Fast neutron interaction	^58^Co (1)	^59^Co (n, 2n) ^58^Co	1.94 × 10^−1^	8.30 × 10^−1^ b at E_n_ = 17 MeV
	^58^Co (2)	^58^Ni (n, p) ^58^Co		6.40 × 10^−1^ b at E_n_ = 9.5 MeV
	^54^Mn	^54^Fe (n, p) ^54^Mn	8.55 × 10^−1^	5.10 × 10^−1^ b at E_n_ = 10 MeV
	^40^K	^40^Ca (n, p) ^40^K	1.28 × 10^9^	6.30 × 10^−1^ b at E_n_ = 8 MeV
Thermal neutron interaction	^59^Fe	^58^Fe (n, γ) ^59^Fe	1.22 × 10^−1^	9.21 × 10 b at E_n_ = 360 eV
	^152^Eu	^151^Eu (n, γ) ^152^Eu	1.35 × 10^1^	6.76 × 10^5^ b at E_n_ = 0.01 meV
	^154^Eu	^153^Eu (n, γ) ^154^Eu	8.59 × 10^0^	1.86 × 10^4^ b at E_n_ = 0.01 meV
	^134^Cs	^133^Cs (n, γ) ^134^Cs	2.06 × 10^0^	1.58 × 10^3^ b at E_n_ = 22.5 eV
	^65^Zn	^64^Zn (n, γ) ^65^Zn	6.70 × 10^−1^	5.88 × 10 b at E_n_ = 281 eV
	^60^Co	^59^Co (n, γ) ^60^Co	5.27 × 10^0^	1.87 × 10^3^ b at E_n_ = 0.01 meV
Proton interaction	^56^Co	^56^Fe (p, n) ^56^Co	2.11 × 10^−1^	-

## Data Availability

Not applicable.
